# Human Immunodeficiency Syndromes Affecting Human Natural Killer Cell Cytolytic Activity

**DOI:** 10.3389/fimmu.2014.00002

**Published:** 2014-01-21

**Authors:** Hyoungjun Ham, Daniel D. Billadeau

**Affiliations:** ^1^Department of Immunology, College of Medicine, Mayo Clinic, Rochester, MN, USA; ^2^Division of Oncology Research and Schulze Center for Novel Therapeutics, College of Medicine, Mayo Clinic, Rochester, MN, USA

**Keywords:** NK cells, primary immunodeficiency, NK cell cytotoxicity, cytotoxic lymphocytes, cytotoxic synapse, lytic granules

## Abstract

Natural killer (NK) cells are lymphocytes of the innate immune system that secrete cytokines upon activation and mediate the killing of tumor cells and virus-infected cells, especially those that escape the adaptive T cell response caused by the down regulation of MHC-I. The induction of cytotoxicity requires that NK cells contact target cells through adhesion receptors, and initiate activation signaling leading to increased adhesion and accumulation of F-actin at the NK cell cytotoxic synapse. Concurrently, lytic granules undergo minus-end directed movement and accumulate at the microtubule-organizing center through the interaction with microtubule motor proteins, followed by polarization of the lethal cargo toward the target cell. Ultimately, myosin-dependent movement of the lytic granules toward the NK cell plasma membrane through F-actin channels, along with soluble *N*-ethylmaleimide-sensitive factor attachment protein receptor-dependent fusion, promotes the release of the lytic granule contents into the cleft between the NK cell and target cell resulting in target cell killing. Herein, we will discuss several disease-causing mutations in primary immunodeficiency syndromes and how they impact NK cell-mediated killing by disrupting distinct steps of this tightly regulated process.

## Introduction

Natural killer (NK) cells comprise 5–15% of human peripheral blood lymphocytes and play an important role in the clearance of virally infected cells, as well as the elimination of cancer cells (1, 2). The main population of NK cells differentiates from hematopoietic stem cells in the bone marrow, and fully matures as CD56^+^CD16^+^ NK cells in secondary lymphoid organs (3). While several recent studies have elegantly demonstrated the presence of a memory immune response in NK cells, previously only attributed to T and B cells of the adaptive immune system (4, 5), NK cells are generally recognized as innate immune cells by the fact that their receptors do not undergo DNA rearrangement processes. Instead NK cells express various germline-encoded activating and inhibitory NK receptors on their cell surface (6–8). Integrated signaling from both activating and inhibitory receptors is thought to enable NK cells to distinguish healthy “self” from infected or stressed unhealthy or “non-self” cells (9, 10). Therefore, activation of NK cells can be achieved via the ligation of a number of activating receptors with their corresponding ligand on the target cell (natural cytotoxicity). In addition, NK cells are also able to detect antibody-coated targets through the low affinity Fc receptor CD16 (FcγRIIIA) and thereby mediate antibody-dependent cellular cytotoxicity (ADCC) (11, 12). The main consequence of NK cell activation is the direct killing of bound unhealthy target cells through the release of perforin and granzymes from a preformed secretory lysosome known as the lytic granule (13, 14). In addition, NK cells can affect the overall immune response through the secretion of chemokines and cytokines (3, 15–18) as well as through direct interaction with other immune cells (2, 5).

Research conducted over the past quarter of a century has uncovered the process of NK cell development, identified and demonstrated the function of activating and inhibitory receptors, and provided a wealth of information regarding the signaling pathways that are linked to these receptors and the proteins that are critical to the delivery of the lethal cargo to the target cell. Significantly, the study of human primary immunodeficiency syndromes (PIDs) has provided invaluable knowledge and insight regarding the role of NK cells in the immune system, and in addition has also resulted in the identification of genes whose protein products regulate distinct steps that are critical to the development of cellular cytotoxicity by this population of innate immune cells. Among the PIDs, several NK cell deficiencies have been identified including rare isolated NK cell deficiencies, where there is total absence or very low number of NK cells in the peripheral blood, which is known as classical natural killer cell deficiency (CNKD) (19, 20). MCM4 and GATA2 are the only two genes identified thus far in which mutations are linked to CNKD, however there remain several cases of CNKD where the underlying genetic cause has not been identified (20). Clearly, sequencing of the genomes in patients with CNKD, which are not caused by mutation of either MCM4 or GATA2, will undoubtedly reveal other genes that play an important role in NK cell development or survival.

The term NK cell deficiency does not just mean an absence of NK cells in the immune system; it also includes the absence of specific NK cell effector functions, like cytotoxicity, despite the presence of normal NK cell numbers. This has been termed functional NK cell deficiency (FNKD) (19, 20). FNKDs have provided us with very valuable insights into the roles of specific NK cell functions in immunity as well as essential proteins required for NK cell effector activities, when the responsible gene has been identified. So far, the only known human genetic mutation leading to FNKD is in the *FCGR3A* gene, which encodes CD16 (20, 21). Similar to patients of CNKD, patients carrying homozygous missense mutations in CD16 (L66H) showed susceptibility to human papilloma virus (HPV) or herpesviridae members. In contrast to CKND, these patients had normal numbers of NK cells, but unexpectedly showed normal ADCC, whereas natural cytotoxicity was defective (20). The fact that the altered amino acid found in these patients is located outside of the immunoglobulin domain (Ig domain) responsible for IgG binding (22) suggests why ADCC of NK cells from the patients is normal. In addition, novel co-stimulatory roles of CD16 mediated by the distal Ig domain of CD16 (23) provided important insights that might explain why the patients’ NK cells showed defective natural cytotoxicity.

Lastly, there are several additional human PIDs that demonstrate defects in NK cell numbers and effector functions. Since many immune cells other than NK cells are also affected, there are additional complications and difficulties in understanding the complex immunological roles of NK cells in these diseases. However, the identification of specific gene mutations has illuminated molecular pathways that are important for NK cell development and effector functions, which are also shared in other immune cell types. In this review, we will specifically focus on PIDs where the mutated gene products impact the intracellular pathways that regulate the development of NK cell-mediated cytotoxicity (Table 1). For detailed discussions about human diseases involved in NK cell development and differentiation, NK cell signaling, or other NK cell effector functions, the reader is referred to other excellent reviews on these topics (19–21, 24).

**Table 1:** **Human primary immunodeficiency syndromes with defective NK cell cytotoxicity**.

NK cytotoxicity process	Disease	Gene mutated	Protein affected	NK cell defects in cytotoxicity	Rescued by IL-2
Lytic granule biogenesis	Familial hemophagocytic lymphohistiocytosis type 2 (FHL2)	*PFR1*	Perforin	Exocytosis of lytic granules is normal, but no cytotoxicity is achieved due to absence of pore-forming molecule	No
	Papillon–Lefèvre syndrome (PLS)	*CTSC*	Cathepsin C	Granzyme B in lytic granules is not fully processed, causing defective cytotoxicity	Yes
	Hermansky–Pudlak syndrome type 2 (HPS2)	*AP3B1*	β3A-subunit of adaptor protein 3	Enlarged lytic granules impaired movement along microtubules? Less perforin in lytic granules?	?
	Chediak–Higashi syndrome (CHS)	*CHS1/LYST*	CHS1/LYST	Enlarged lytic granules. Impaired exocytosis of lytic granules (unknown cause)	?
Adhesion to target cells	Leukocyte adhesion deficiencies type 1 (LAD-I)	*ITGB2*	β2-subunit of integrin (CD18)	Impaired adhesion to target cells. Impaired polarization	Yes
	Leukocyte adhesion deficiencies type 3 (LAD-III)	*FERMT3*	Kindlin-3	Impaired adhesion. Impaired specific activating receptor-mediated cytotoxicity, but normal natural cytotoxicity	?
F-actin rearrangement	Wiskott–Aldrich syndrome (WAS)	*WASP*	WASP	Impaired adhesion to target cells. Impaired reorganization of F-actin and integrins. Impaired polarization of lytic granules.	Yes
	WASP-interacting protein (WIP) deficiency	*WIPF1*	WIP	Reduced surface expression of some NK activating receptors. No detectable WASP.	No
	Dedicator of cytokinesis 8 (DOCK8) deficiency	*DOCK8*	DOCK8	Impaired adhesion to target cells. Impaired reorganization of F-actin and integrins. Impaired polarization of lytic granules.	No
Polarization of lytic granules toward CS	MYH9-related diseases (MYH9-RD)	*MYH9*	Myosin IIA	Impaired exocytosis of lytic granules	?
Fusion of lytic granules into PM	Griscelli syndrome type 2 (GS2)	*RAB27A*	RAB27A	Impaired exocytosis of lytic granules	Yes
	Familial hemophagocytic lymphohistiocytosis type 3 (FHL3)	*UNC13D*	Munc13-4	Impaired exocytosis of lytic granules	No
	Familial hemophagocytic lymphohistiocytosis type 4 (FHL4)	*STX11*	Syntaxin-11	Impaired exocytosis of lytic granules	Yes
	Familial hemophagocytic lymphohistiocytosis type 5 (FHL5)	*STXBP2*	Munc18-2	Impaired exocytosis of lytic granules	Yes

*? = Unknown or equivocal*.

## Biogenesis of Lytic Granules and Their Maturation

Cell-mediated killing by NK cells and cytotoxic T lymphocytes (CTLs) is achieved by directed release of lytic granules toward bound target cells. Lytic granules are dual-function organelles that exhibit characteristics of a degradative lysosome, but can also secrete granule constituents for cell-mediated killing and are therefore often referred to as a secretory lysosome. This specialized organelle is mostly observed in hematopoietic lineage cells, but it is also found in melanocytes that produce pigment proteins called melanins (25, 26). Both secretory lysosomes and conventional lysosomes are acidic organelles (pH 5.1–5.4), morphologically similar, and contain proteins like acid hydrolases that are required for their degradative function as well as receptor proteins including members of the lysosomal-associated membrane protein (LAMP) family. On the other hand, the main distinction of secretory lysosomes from conventional lysosomes is that secretory lysosomes undergo a regulated secretion process and they contain additional contents that are cell-type-specific. In the case of NK cells and CTLs, these specific secreted contents include perforin and granzymes, which are essential for cytotoxic activity. On the other hand, the main secreted proteins of melanocytes are melanins, which are pigments responsible for skin color. Cells containing secretory lysosomes use common secretory machineries, but cell-type-specific secreted contents enable each cell type to perform different effector functions. This is why human genetic diseases such as Chediak–Higashi syndrome (CHS) and type 2 Hermansky–Pudlak syndrome (HPS), caused by impaired function of the secretory lysosome, are characterized not only by severe immune deficiencies including defective cytotoxicity by NK cells and CTLs but also by hypopigmentation due to defects in melanosome formation and excessive bleeding due to the absence of dense granules in platelets (26–28).

In contrast to CTLs, which need to be activated, the genes encoding perforin and granzymes are constitutively transcribed in NK cells thus allowing them to immediately kill an infected or stressed cell upon initial contact and without the need for additional gene expression (29, 30). Perforin is essential for NK cell-mediated cytotoxicity, since it is the only molecule that delivers apoptosis-inducing granzymes into the target cell. Perforin is initially synthesized as an inactive precursor in the ER and undergoes multiple posttranslational modifications including proteolysis and glycosylation during its transit to the Golgi and finally to the lytic granule (31). However, the exact pathway that sorts perforin from the *trans*-Golgi network (TGN) into lytic granules is unresolved. After arriving into lytic granules, perforin is further processed by cathepsin L to develop into a mature form. In the case of granzymes, five different granzymes, granzyme A, B, H, K, and M, are expressed in human NK cells and CTLs. They are synthesized as pro-enzymes and modified to have a mannose-6-phosphate moiety in the *cis*-Golgi, which facilitates their trafficking to the endosome by the mannose-6-phosphate receptor (M6PR) before finally arriving into the lytic granule (32, 33). In lytic granules, granzymes are further processed into an active form by various cathepsins (34–36). Granzymes are serine proteases and each granzyme has different substrate specificity and induces apoptosis in the target cell in both caspase-dependent and caspase-independent manners (37, 38).

An important question to be asked is: How do cytotoxic lymphocytes protect themselves from these dangerous molecules? Significantly, the activity of perforin is achieved by the membrane attack complex/perforin (MACPF) domain, which has cytolytic activity, and a C2 domain that enables binding to membrane in a calcium-dependent manner (37, 39, 40). Importantly, the low level of Ca^2+^ in lytic granules maintains perforin in an inactive conformation. In addition, calreticulin, another component of the lytic granule, inhibits perforin activity (41), and the acidic environment of lytic granules provides protection to NK cells since the activity of both perforin and granzymes are inhibited at this low pH. Furthermore, the low pH environment favors association of serglycin, a proteoglycan matrix found within lytic granules, with both perforin and granzymes. This interaction keeps both perforin and granzymes in an inactive state until they are secreted into a neutral pH environment (42). Interestingly, the mechanism by which perforin facilitates the transport of granzymes into the target cell is still unclear (37, 40). According to the prevailing model, perforin is released into the cytotoxic synaptic cleft where it binds calcium, oligomerizes on the target membrane, and forms pores. The high concentration of calcium as well as the neutral pH at the interface between NK cells and target cells relieves perforin from its inhibited conformation and triggers its activity (40). It is assumed that the established pore is large enough for granzymes to get into target cells by simple diffusion (21, 37, 40). Last but not least, how do NK cells prevent themselves from potential self-destruction from released cytotoxic components? Cell surface expression of cathepsin B has been suggested to provide protection of cytotoxic lymphocytes by cleaving perforin (43). In addition, it was recently shown that the surface expression of LAMP-1 (CD107a) also protects NK cells from degranulation-associated damage by inhibiting binding of perforin to NK cells (44). Therefore, it seems there exist multiple layers of protection at the final degranulation step that ensure unidirectional cytotoxicity. In the following paragraphs, we will focus our discussion on PIDs with impaired NK cell cytotoxicity caused by: (1) defects in the contents of lytic granules and (2) impaired biogenesis and maturation of lytic granules.

### PIDs affecting contents of lytic granules

#### Familial hemophagocytic lymphohistiocytosis type 2

Familial hemophagocytic lymphohistiocytosis type 2 (FHL2) is an autosomal recessive disorder comprising 13–58% of all FHL cases and is caused by mutation of the *PFR1* gene, which encodes perforin (45). Most of the mutations identified in FHL2 patients occur within regions critical for perforin maturation, or impair proper folding, oligomerization, or Ca^2+^-mediated membrane binding (31, 46). Interestingly, each mutation can dramatically impact the level of mature perforin, ranging from absent to normal. Additionally, the intrinsic activities of the mutated perforin correlate with the age of FHL onset and the severity of the disease (47–52). Significantly, the inability of the mutated perforin to form pores on target cell membranes results in the absence of cytotoxic function of NK cells from FHL2 patients. Perforin loss did not affect the level of other lytic granule components (granzymes and cathepsins) or the steps leading to lytic granule polarization and membrane fusion (45, 53). Therefore, the normal degranulation (examined by surface expression of CD107) observed in NK cells from FHL2 patients provides us an important criterion to distinguish FHL2 patients from FHL patients caused by mutation of other genes (53). In many cases, FHL2 patients usually further develop other diseases including leukemia, juvenile rheumatoid arthritis, and macrophage activation syndrome (48, 54–61), suggesting an important role for perforin and cytotoxic activity mediated by NK cells and CD8^+^ T cells in limiting or preventing these diseases. In addition, the non-redundant role of perforin activity in cellular cytotoxicity suggests the intervention of perforin activity as a potential therapeutic target in human diseases caused by abnormal cytotoxicity of cytotoxic lymphocytes (52).

#### Papillon–Lefèvre syndrome

Papillon–Lefèvre syndrome (PLS) is a rare autosomal recessive disease caused by mutation of the gene encoding cathepsin C, *CTSC* (62–64). This disease is clinically characterized by palmoplantar keratosis, early onset of severe periodontitis, and susceptibility to viral infections. Cathepsin C is a lysosomal cysteine protease, which is responsible for the processing of granzyme A and B (36, 65). Consequently, NK cells from PLS patients primarily contain immature granzyme B, and hence, their NK cells show impaired cytotoxic activity (34). Interestingly, the impaired processing of granzyme B as well as the defective cytotoxicity could be restored by treatment of interleukin-2 (IL-2), suggesting that an IL-2 signaling pathway is able to process granzyme B in a cathepsin C-independent manner (34, 66).

### PIDs affecting biogenesis and maturation of lytic granules

#### Hermansky–Pudlak syndrome type 2

Hermansky–Pudlak syndrome is an autosomal recessive disease clinically characterized by oculocutaneous albinism and excessive bleeding (67, 68). Among the currently identified nine different types of HPS, Hermansky–Pudlak syndrome type 2 (HPS2) is the only type known to cause immunodeficiency in addition to other clinical symptoms (67, 69, 70). HPS2 is caused by mutation of the *AP3B1* gene that encodes the β3A-subunit of adaptor protein 3 (AP3), which is part of a heterotetrameric protein complex (67, 71). Mutations leading to the loss of the β3A-subunit affect the stability of other subunits and thereby induce loss of the entire complex (67). The AP3 complex mediates sorting of integral membrane proteins from the endosome and TGN to the lysosome and secretory lysosome (72, 73). Considering the ubiquitous expression of AP3, it is very interesting to note that its absence causes severe functional defects only in cells with secretory lysosomes, indicating that AP3 is required for the biogenesis of secretory lysosomes and/or in sorting of secretory lysosome-specific proteins.

In the case of melanocytes, the AP3 complex was shown to be responsible for sorting of tyrosinase, a protein required for melanin synthesis, into the melanosome (74). Additionally, AP3-deficiency was shown to cause defective cytotoxicity of both NK cells and CTLs (17, 75–77). While perforin levels in CTLs have been reported to be within the normal range in some patients with HPS2 (76), NK cells from two siblings with HPS2 showed less intracellular perforin levels compared to NK cells from healthy controls (75), suggesting that the defective NK cell-mediated cytotoxicity observed in these patients is at least partially a result of diminished perforin. Considering the fact that these patients had similar mRNA expression of perforin, correct sorting of existing perforin into lysosomes and the existence of mature perforin in patient NK cells, perforin biosynthesis seems to be independent of AP3 complex (75). Yet it remains possible that the AP3 complex is involved in sorting of proteins critical for perforin stability once perforin has made it into the lytic granule. This discrepancy between CTLs and NK cells from these patients might be due to further cell-type-specific roles of AP3 between NK cells and CTLs since lytic granule biogenesis is known to be different between these two cell types; lytic granules are preformed in resting NK cells, whereas lytic granules are generated after activation in CTLs (78–80). Clearly more patients need to be accumulated to better understand these different observations. It is also interesting to note that enlarged lytic granules were observed in CTLs of HPS2 patients (77). Further elucidation of the AP3 complex target proteins required for lytic granule sorting as well as other potential roles of the AP3 complex in the biogenesis of lytic granules will be able to answer these questions.

Lastly, Clark et al. suggested that the AP3 complex might also play a more direct role(s) in the cytotoxic process of CTLs. They observed that lytic granules from AP3-deficient CTLs fail to cluster around the microtubule-organizing center (MTOC) when conjugated with appropriate target cells (77). This seems to be due to a failure of lytic granules to move along the microtubules or an inability to attach to microtubules. In addition, it is not clear whether this observed defect is a result of a direct role for AP3 in mediating lytic granule clustering at the MTOC, or due to a sorting failure of a protein(s) critical for attachment and/or movement of lytic granules along microtubules. Definitely, future studies elucidating the mechanism resulting in the phenotypes of AP3-deficiency in CTLs and NK cells will be very exciting.

#### Chediak–Higashi syndrome

Chediak–Higashi syndrome is another disease caused by defective lytic granule generation in NK cells and CTLs. The disease is inherited in an autosomal recessive manner and the main clinical symptoms of CHS are partial albinism and recurrent infections in the lung and skin. Mild coagulation defects and varying degrees of neurologic dysfunctions are also commonly observed in CHS patients. Although patients are often able to clear bacterial infections with the help of antibiotics, they instead develop an accelerated phase of hemophagocytic lymphohistiocytosis; seen by infiltration of macrophages and lymphocytes into the major organs of the body (81). Clinically, CHS and Griscelli syndromes (GSs; discussed in later section) present with identical features. However, at the cellular level, CHS can be distinguished from GS by the fact that CHS cells contain abnormally enlarged lysosomes and secretory lysosomes. Therefore, this cellular feature is used as the key diagnostic criterion of CHS (82–84). In 1996, the responsible gene for CHS was mapped to *CHS1/LYST* gene (*beige* gene in the mouse model of CHS) (85, 86). The *CHS1/LYST* gene is highly conserved in all species, and a unique region at the C-terminal end of the protein called the BEACH (Beige and Chediak) motif defines a family of proteins homologous to the CHS1/LYST protein. Our current understanding suggests that all BEACH family proteins participate in vesicle trafficking (83, 87).

CHS1/LYST proteins are expressed at very low levels in all cell types, and are involved in maintaining the morphology of lysosomes and secretory lysosomes (83). However, similar to HPS2, only cells with secretory lysosome function are impaired (83, 88). In fact, although few in number, giant lytic granules are observed in NK cells and CTLs from CHS patients, and defects in cytotoxicity of both cell types have been reported from many studies (19, 21). In the case of NK cells from CHS patients, their numbers are within normal range, but the absence of CHS1/LYST causes defects in natural cytotoxicity as well as ADCC (89–95). NK cells from CHS patients are able to efficiently bind target cells suggesting that the defect in NK cell-mediated cytotoxicity is due to problems in the actual cytotoxicity process. However, detailed molecular roles of CHS1/LYST in NK cell-mediated cytotoxicity have not been reported to date. Interestingly, treatment with interferons (IFNs) increase the cytotoxic capacity of NK cells from CHS patients (92, 93, 96), suggesting that IFN signaling is able to rescue cytotoxicity defects of CHS NK cells, at least partially.

Most of our knowledge regarding the cellular functions of CHS1/LYST protein were obtained from studies of CTLs from CHS patients. Unexpectedly, while giant lysosomal organelles were observed in the patients’ cells, the biogenesis, processing, and expression level of lytic proteins such as perforin and granzymes were normal, as were their sorting into lytic granules after activation. However, as time went on, the lytic granules began to fuse together becoming giant organelles (97, 98). Based on these observations, it is likely that the CHS/LYST protein either prevents abnormal fusion between lysosomal organelles or separates lysosomal membranes after normal fusion events, thus regulating lysosomal size. Significantly, CTLs from CHS patients were unable to secrete their granules in response to TCR ligation (97), suggesting that the cytotoxicity defects observed in NK cells and CTLs from CHS patients result from an exocytosis defect. Further studies elucidating the roles of CHS1/LYST in lysosomal fusion and exocytosis as well as the roles of other BEACH family proteins will provide clearer insight into the NK and CTL phenotypes observed in patients as well as the biogenesis of lytic granules and melanosomes.

## Adhesion of NK Cells to Target Cells

**Figure 1:**
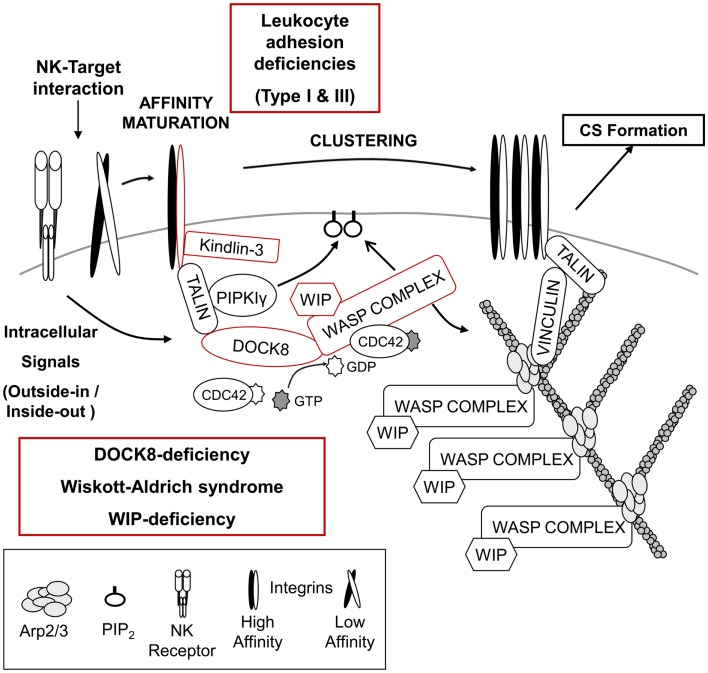
**Regulation of NK – target cell adhesion and generation of F-actin at the cytotoxic synapse**. Initial signals from the NK – target interaction recruit multiple proteins responsible for integrin-mediated adhesion and high affinity maturation as well as the accumulation of F-actin at the cytotoxic synapse (CS). Absence of either the integrin β2-subunit (CD18) or its regulator, Kindlin-3, results in leukocyte adhesion deficiency type I or type III, respectively. NK cells from both diseases present defective cytotoxicity due to failure of efficient target binding and/or defective NK cell activation. The DOCK8–WASP complex is also recruited to the NK-target interface, and a “DOCK8–CDC42– WASP” pathway is likely responsible for F-actin reorganization, which might facilitate integrin-mediated adhesion and provide the F-actin meshwork critical for organization of the CS. Absence of either DOCK8 or WASP results in primary immunodeficiency disorders and NK cells from these patients show impaired NK cytotoxic activity (see text for details).

Natural killer cells rapidly accumulate at the site of inflammation through the stimulation of inflammatory chemokine receptors and adhesion molecules expressed on their cell surface (1). Although the roles of weak adhesion molecules like selectin family members have not been directly examined in NK cell – target cell binding, it is generally appreciated that the first step toward the development of NK cell cytotoxicity is weak integrin-mediated adhesion to target cells (99). Signals from this weak adhesion, along with the initial signaling resulting from the engagement of activating/inhibitory receptors on the NK cell with corresponding ligands on the target cell, determines whether the NK cell should detach (strong inhibitory signal) or establish a firm adhesion (strong activation signals) leading to the formation of the cytotoxic synapse (CS). Along with high affinity interactions toward their ligands, integrins were also observed to cluster at the CS of NK cells, strengthening adhesion (avidity) (100–102) and promoting the scanning of target cells (Figure 1) (11, 12, 99). In addition to being an important mediator of cell–cell adhesion, integrins can serve as co-stimulatory molecules enhancing cytotoxicity signals from other activating NK receptors (103–105), as well as promoting lytic granule polarization following ligand engagement (104, 106–108).

### Leukocyte adhesion deficiency type I

Leukocyte adhesion deficiencies (LAD) are autosomal recessive diseases caused by mutations in the genes encoding adhesion molecules or molecules critical for processing/activation of adhesion molecules. Leukocyte adhesion deficiency type I (LAD-I) is the most common type of LAD, and is caused by mutation in the gene encoding the β2-subunit of the integrin (CD18), *ITGB2* (Figure 1) (109). A variety of mutations have been found in *ITGB2*, which can impact the expression of CD18 as well as its ability to bind a ligand (110). The main clinical symptoms of these patients are recurrent bacterial and fungal infections in the skin and mucosa, absence of pus formation at the infection sites, and impaired healing after infection. An abnormally high number of leukocytes in the blood are also a common feature of this disease, indicative of inefficient recruitment to sites of inflammation (19, 110). Supporting the essential role of integrins in mediating cytotoxicity signaling, NK cells from LAD-I patients showed defective natural cytotoxicity as well as ADCC compared to control NK cells (111–113). Although the defective cytotoxicity of patients’ NK cells seems to be mainly due to impaired adhesion to target cells, it was recently suggested that LFA-1 (CD11a/CD18) might be required for clustering of lytic granules to the MTOC after target cell binding (114). However, further studies are required to clearly conclude that impaired lytic granule convergence observed in NK cells of LAD-1 patients is due to the absence of LFA-1. It remains possible that the defect observed in LAD-I NK cells might result from impaired/delayed signaling from other NK receptors caused by defective adhesion to target cells. One study suggested that the cytotoxicity defects in LAD-I NK cells could be rescued at least partly via activation with IL-2 (115). In this regard, it is very interesting to note that activation of NK cells with only IL-2 treatment promoted clustering of lytic granules (114). How IL-2 signaling is able to rescue cytotoxicity of NK cells (adhesion to target cells or clustering of lytic granules) is unclear and will require further investigation.

### Leukocyte adhesion deficiency type III

Leukocyte adhesion deficiency type III (LAD-III) is caused by mutation of the *FERMT3* gene, which encodes Kindlin-3 (110). Kindlin-3 is expressed only in cells of the hematopoietic lineage (116), and like talin, regulates integrin-mediated adhesion by binding directly to the integrin β-chain (Figure 1) (117, 118). Patients with LAD-III have severe recurrent infections, leukocytosis, and persistent bleeding (110). As expected from the main role of Kindlin-3 in integrin-mediated adhesion, defective integrin activation was observed in platelets, lymphocytes, and polymorphonuclear leukocytes (PMNs) derived from LAD-III patients (119–125). Recently, functions of NK cells from a single female infant patient were examined (126). Interestingly, specific activating receptor-mediated cytotoxicity was defective, whereas natural cytotoxicity mediated by multiple NK receptors was normal. It was suggested that Kindlin-3 might function by lowering the signaling threshold required for NK cell activation. Clearly, more patients will be needed to further define its role in NK cell-mediated cytotoxicity. In addition, further insights into the cellular roles of Kindlin-3 in inside-out/outside-in signaling by integrins as well as elucidation of the relationship of Kindlin-3 and talin in integrin regulation will be very helpful in understanding LAD-III.

## Actin Reorganization at the Cytotoxic Synapse

Essential signaling molecules like NK activating/inhibitory receptors are accumulated at the center of the interface known as the central supramolecular activation cluster (cSMAC), whereas F-actin and integrins are localized at the periphery of the interface forming a ring-shaped peripheral supramolecular activation cluster (pSMAC). Among these distinct molecular patterns at the CS, F-actin accumulation at the pSMAC is thought to be one of the early and critical events in NK cell-medicated cytotoxicity, since clustering of NK receptors as well as adhesion molecules are observed to be dependent on F-actin polymerization (13, 99, 101). Two primary immunodeficiencies causing defective F-actin accumulation at the CS of NK cells have provided some clues on how F-actin accumulation might be regulated at the CS.

### Wiskott–aldrich syndrome

Wiskott–Aldrich syndrome (WAS) is an X-linked primary immunodeficiency disorder characterized by recurrent infections, prolonged bleeding, eczema, thrombocytopenia, and impaired cellular and humoral immunity (127, 128). The disease is caused by mutations of the *WAS* gene, which encodes the hematopoietically expressed WAS protein (WASP) (129, 130). Depending on the disease severity and type of mutation, WAS is further subcategorized as X-linked thrombocytopenia (XLT) or X-linked neutropenia (XLN) (127, 128). WASP is the founding member of the WASP superfamily of F-actin nucleation promoting factors (NPFs), which stimulate the formation of branched F-actin via the ubiquitously expressed Arp2/3 complex (Figure 1) (131). All members of the WASP superfamily contain a C-terminal verprolin-connecting-acidic (VCA) domain that interacts with profilin-G-actin and the Arp2/3 complex (127, 128, 132). WASP is thought to play diverse roles in immune cells including formation of the immunological synapse (IS), migration, and phagocytosis (127, 128, 132). WASP-interacting protein (WIP; discussed in the next section) constitutively stabilizes WASP in an auto-inhibited conformation (133) in which the VCA domain is stabilized through an interaction with the basic region (BR) and CDC42/RAC GTPase binding domain (GBD). Cell stimulation results in the cooperative interaction of the BR with phosphatidylinositol (4,5)-biphosphate (PIP_2_) and active CDC42 (CDC42–GTP) with the GBD, thereby leading to the release of the bound VCA domain so that it can bind the Arp2/3 complex and promote branched F-actin generation (134–138).

Wiskott–Aldrich syndrome protein was observed to localize at the pSMAC of the CS along with F-actin in target-bound NK cells, and the roles of WASP in NK cell-mediated cytotoxicity have been examined using NK cells from WAS patients (101, 139, 140). Although the proportion of NK cells in the blood was within the normal range or higher in WAS patients, WAS NK cells showed defective natural cytotoxicity as well as ADCC (139, 140). Impaired cytotoxicity of WAS NK cells was due to inefficient conjugate formation with target cells, a failure in F-actin accumulation at the cell–cell contact site, clustering of integrins and CD2, and polarization of lytic granules at the CS (Figure 1) (101, 139, 140). Interestingly, *ex vivo* as well as *in vivo* treatment with IL-2 was able to rescue impaired cytotoxicity of WAS NK cells (139, 141). IL-2 treatment of WAS NK cells restored target binding efficiency, F-actin accumulation, and polarization of lytic granules to the CS. The IL-2-mediated rescue of the defect in WAS NK cells was found to be independent of WASP function, but dependent on another WASP family member, WAVE2 (141). These findings suggest that there exists two distinct pathways for Arp2/3-generated F-actin reorganization at the CS in NK cells, and future studies should enable the use of IL-2 as a therapy to improve clinical symptoms of WAS including severe herpes simplex virus (HSV) infection caused by defective NK cell-mediated cytotoxicity.

Activation of NK cells by target cells or stimulation of CD16 was shown to activate CDC42 in NK cells (139), and accumulating observations suggest that the spatiotemporal activation of CDC42 in NK cells is important in CS formation and cytotoxic activity (142, 143). Therefore, based on current knowledge, NK cell activation results in the generation of active CDC42 at the CS, and this leads to the activation of WASP. Activated WASP will then contribute to F-actin reorganization at the CS, which will mediate the clustering of CD2, integrins, and NK receptors. However, there are still many important questions to be answered regarding the role and regulation of WASP in NK cell-mediated cytotoxicity. For example: (1) What are the upstream signaling pathways that lead to the activation of CDC42 and the generation of PIP_2_ that are required for WASP activation at the CS? (2) How is WASP accumulated at the CS after the stimulation of NK cells? and (3) How does WASP mediate the polarization of lytic granules toward the CS? Future studies aimed at addressing these and other important questions will provide insight into how WASP is regulated and mechanistic insight into its role in the development of NK cell-mediated killing.

### WASP-interacting protein deficiency

Deficiency of WASP-interacting protein was recently identified in a female infant patient (144). The patient had a homozygous mutation in the *WIPF1* gene and both of her parents were heterozygous for the same mutation, suggesting that the disease is inherited in an autosomal recessive manner. WIP is ubiquitously expressed in humans, although higher levels are observed in hematopoietic cells (145). The clinical features were very similar to those observed in WAS patients, including recurrent infections, thrombocytopenia, eczema, and immunodeficiency. This is not surprising as WIP is thought to maintain the stability of WASP and regulate WASP activity (Figure 1) (145, 146). Consistent with this, WASP protein was not detected in the patient’s T cells, despite normal *WAS* mRNA expression. However, several WASP-independent functions of WIP have been observed in NK cells. First of all, WIP was shown to be essential for formation of a multiprotein complex composed of WIP, WASP, actin, and myosin IIA after NK cell activation (147). WIP was also found to associate with the lytic granules in NK cells and WIP-depleted NK cells failed to polarize their lytic granules toward the CS (148). In addition, the expression level of major NK activating receptors (NKG2D, NKp30, and NKp46) was lower on WIP-deficient NK cells compared to control NK cells. These findings also suggest that patients with WAS symptoms cannot be simply diagnosed based on WASP expression at the protein level, and will require sequence analysis of the *WAS* and *WIPF1* genes to distinguish between WAS and WIP deficiency.

### Dedicator of cytokinesis 8 deficiency

Another PID that causes defective F-actin accumulation at the CS of NK cells is the loss of dedicator of cytokinesis 8 (DOCK8). DOCK8 is a member of the DOCK180 family of guanine nucleotide exchange factors (GEFs) that mediate activation of Rho family proteins and contribute to multiple cellular processes including cell migration and phagocytosis (149–151). DOCK8 deficiency is a relatively recently identified primary combined immunodeficiency that shows an autosomal recessive pattern of inheritance (152, 153). The main clinical symptoms of these patients are recurrent infections in the lung and skin, high IgE levels in the serum, and severe allergies (152–154). The roles of DOCK8 in human NK cells have been examined recently using NK cells from DOCK8-deficient patients as well as primary human NK cells and human NK cell-lines depleted of DOCK8 by RNAi (155, 156). DOCK8-deficient/depleted NK cells showed defective natural cytotoxicity and specific activating receptor-mediated NK cytotoxicity, suggesting that functional defects of NK cells in DOCK8-deficient patients could be part of the cause for the frequent sinopulmonary infections observed in these patients (152–154, 157). Defective accumulation of F-actin and clustering of integrins at the IS were also observed in DOCK8-deficient/depleted NK cells as well as B cells and CD8^+^ T cells from DOCK8-mutant mice (155–158). Polarization of lytic granules toward the CS was also defective in DOCK8-deficient/depleted NK cells (155, 156). Mechanistically, DOCK8 was found to interact with WASP and talin, and to mediate their localization to the CS of NK cells (Figure 1) (155). Significantly, DOCK8 was found to have GEF-specificity toward CDC42 (155, 159). These findings provide an important link between the signaling pathways that might activate WASP and may help explain the remarkable similarities between WAS and DOCK8 deficiency. In fact both diseases share many clinical symptoms [recurrent infections by certain types of viruses, and high IgE levels] (127, 128, 132, 152–154, 157), as well as phenotypes observed in NK cells [defective cytotoxic activity, integrin-mediated adhesion, F-actin accumulation, and polarization of lytic granules (101, 139, 140, 155, 156)].

Surprisingly, the addition of IL-2 was unable to rescue the cytotoxic defects seen in DOCK8-deficient NK cell suggesting that DOCK8 is likely involved in the regulation of other signaling pathways in addition to its role in localizing and potentially activating WASP. However, based on the current findings, it is very tempting to hypothesize that DOCK8 mediates the recruitment of WASP to the CS of NK cells after activation where it can generate active CDC42 thus leading to the activation of WASP (Figure 1). Another DOCK8-interacting partner, talin, associates with phosphatidylinositol phosphate kinase type Iγ (PIPKIγ), which produces PtdIns (4,5)P2 (PIP_2_), a molecule required for optimal activation of WASP (134–138, 160–162). The activated WASP is then able to interact with Arp2/3 inducing the generation of a branched F-actin network, and this enhances the clustering of integrins and NK receptors. Future studies testing the hypothesis of the “DOCK8–CDC42–WASP” pathway should provide a clearer picture of the formation of the CS. Lastly, one remaining question regarding the role of DOCK8 and WASP is how they mediate the polarization of lytic granules toward the CS. Based on current knowledge, it is most likely that polarization of lytic granules toward the CS occurs later than F-actin accumulation (101) since cytochalasin D (Cyt D)-treated NK cells also showed defective polarization of lytic granules (140). However, considering the fact that CDC42 is best known for regulating cellular polarity and is responsible for MTOC polarization in T cells, macrophages, and dendritic cells (DCs) (163–167), DOCK8 might regulate MTOC polarization through the activation of other CDC42 effector molecules in addition to regulating the CDC42–WASP pathway leading to F-actin accumulation.

## Polarization of Lytic Granules Toward Cytotoxic Synapse

**Figure 2:**
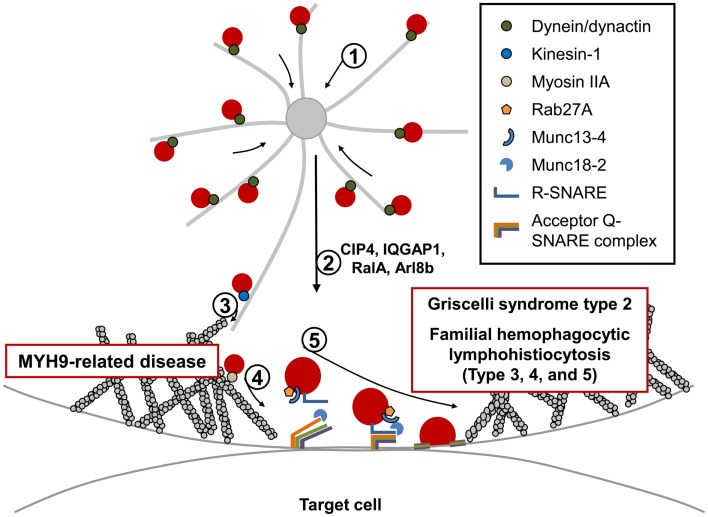
**Fusion and exocytosis of lytic granules during NK cell-mediated cytotoxicity**. Directed release of lytic granules toward bound target cells is a multi-step process: (1) lytic granules are rapidly clustered around the MTOC. This retrograde (minus-end-directed) movement along the microtubules occurs rapidly and is mediated by the dynein/dynactin motor complex. (2) The MTOC, along with accumulated lytic granules, are polarized toward the CS, and several proteins have been identified to be critical for this step. (3) Polarized lytic granules are further delivered toward the CS along the microtubule (anterograde transport) in a kinesin-1-dependent manner. (4) Myosin IIA facilitates the final transit of the lytic granules through the hypodense F-actin meshwork at the CS. NK cells from patients with MYH9-related diseases fail to degranulate. (5) The final fusion of lytic granules with the plasma membrane occurs in an orchestrated manner and requires multiple molecules including Rab27a, its effector protein Munc13-4, SNARE proteins, and their accessory proteins. Mutations in proteins involved in this final fusion step have been found to cause immunodeficiency disorders including Griscelli syndrome type 2, familial hemophagocytic lymphohistiocytosis types 3, 4, and 5, all of which have similar clinical symptoms, and defective lytic granule exocytosis.

Another important step required for NK cell-mediated cytotoxicity is the polarization of lytic granules toward the CS. This event occurs after the rearrangement of F-actin at the CS and requires both F-actin polymerization and microtubule function, since lytic granules fail to polarize toward the CS when either F-actin accumulation or the microtubule network are disrupted (101). As NK cells start to form conjugates with target cells, lytic granules are rapidly clustered around the MTOC (Figure 2) (107). This retrograde, minus-end-directed transport of lytic granules on microtubules is Src kinase-dependent, and mediated by the dynein/dynactin motor complex that constitutively associates with lytic granules and tethers them to the microtubules (107, 114, 168). In conjunction with the clustering of the lytic granules, the MTOC polarizes toward the CS, thereby delivering the lethal cargo to the site of target cell contact (101, 107, 168). Importantly, this process occurs along with maturation of the CS, as it requires F-actin accumulation, rearrangement of NK receptors, and clustering of adhesion molecules.

Current knowledge suggests that activation of extracellular-signal-regulated kinase (Erk), Vav1, and protein tyrosine kinase 2 (PYK2) are required for MTOC polarization in NK cells (169–171). In addition to these molecules, several other proteins have been identified that impact the polarization of lytic granules to the CS (Figure 2). For example, interrupting the cellular function of the CDC42-interacting protein-4 (CIP4) was shown to impair MTOC polarization as well as NK cell-mediated cytotoxicity (172). CIP4 was suggested to link the microtubule and F-actin network at the CS through its ability to interact with microtubules and WASP. In this regard, the scaffold protein IQGAP1 also has the potential to interact with F-actin and microtubules, and silencing of IQGAP1 was also shown to impair NK cell cytotoxicity due to defective MTOC polarization toward the CS (173). However, MTOC polarization and cell-mediated killing were found to be normal in mouse CTLs lacking IQGAP1 (174), highlighting again that distinct mechanisms regulating cell-mediated killing are engaged in these two cell types. The Ral GTPase, RalA, also regulates MTOC polarization toward the CS (175). Very recently, the small GTP-binding protein, Arl8b, was found to interact with KIF5B, the heavy chain of the microtubule plus-end-directed motor kinesin-1, and mediate MTOC polarization in NK cells (176). How and where the kinesin-1 motor is recruited to mediate movement of both the MTOC and lytic granules toward the CS remains unclear. In CTLs, a Rab27a/Slp3/kinesin-1 complex was reported to be critical for final transport of polarized lytic granules along with microtubules toward the CS (Figure 2). Interestingly, polarization of the MTOC toward the CS was normal when kinesin-1 was interrupted in CTLs (177). It will be interesting to analyze these seemingly different roles of kinesin-1 in both NK cells and CTLs. Lastly, although not examined in NK cells, activity of CDC42 might also be essential for MTOC polarization considering similar roles in other immune and non-immune cells (163–167).

Following the clustering of the lytic granules at the MTOC, the MTOC docks near the plasma membrane (PM) facilitating fusion of lytic granules into the secretory cleft formed between the NK cell and target cell (168, 178). Previous studies using conventional confocal microscopy suggested that the lytic granules pass through an actin-cleared region at the center of the CS (101, 102); however, recent approaches using super-resolution microscopy suggest that the secretory region at the center of the CS is not totally absent of F-actin. Instead, a hypodense branched F-actin meshwork exists in the central region of the CS that is rearranged to produce small pores through which the lytic granules travel (179, 180). The previously suggested role of myosin IIA, an actin motor protein, in the final transit of lytic granules through the actin-rich region of the CS are consistent with this new finding and will be discussed in the next section (Figure 2). It still remains to be determined how the F-actin network at the secretory region is regulated to allow lytic granule movement by the myosin motor protein.

### MYH9-related disease

Myosin IIA is an actin-based molecular motor that promotes movement along F-actin and is known to be involved in numerous cellular processes (181). Myosin IIA is a hexameric protein composed of two heavy chains, two regulatory light chains, and two essential light chains. The heavy chain of myosin IIA contains a motor domain at the N-terminal head that binds to F-actin to perform its motor function through ATP hydrolysis, whereas the C-terminal region performs filament formation, cargo binding, and regulatory functions (182–184). In humans, mutations in *MYH9* gene, which encodes the heavy chain, lead to autosomal dominant diseases collectively known as MYH9-related diseases (MYH9-RDs), which include May–Hegglin Anomaly, Sebastian Syndrome, Fechtner Syndrome, and Epstein Syndromes (185, 186). The main clinical features of MYH-RD are macrothrombocytopenia and granulocyte inclusions.

Previous studies using the myosin light chain kinase inhibitor ML-9 (1-[5-chloronaphthalene-1-sulfonyl]-1*H*-hexahydro-1,4-diazepine) inhibited NK cell-mediated cytotoxicity (187), suggesting that myosin IIA would be critical for NK cell effector activity. Consistent with this notion, NK cells from MYH9-RD patients as well as human NK cells that were either inhibited pharmacologically or depleted of myosin IIA function had impaired NK cell-mediated cytotoxicity (188–190). Molecularly, it was found that inhibition or depletion of myosin IIA did not affect the early steps of NK cytotoxicity including target binding, F-actin accumulation, or polarization of lytic granules, but caused impaired degranulation (Figure 2). It was further found that a pool of myosin IIA is associated constitutively with lytic granules, and facilitates the final transit of the lytic granules through the F-actin meshwork at the secretory region of the CS (190). It is important to note that there might be other important roles of myosin IIA in NK cell-mediated cytotoxicity. For example, as mentioned in the previous section, it was shown that myosin IIA and actin are recruited to WIP during NK cell activation (147). Lastly, a large fraction of myosin IIA is found associated with the F-actin at the cell cortex, suggesting that there are likely other functions of myosin IIA in addition to its role in regulating lytic granule trafficking at the CS (147, 188, 190). Future studies regarding other cellular roles of myosin IIA in the NK cytotoxicity and their connection with roles in lytic granule transport will be interesting.

## Fusion of Lytic Granules with the Plasma Membrane

Exocytosis of lytic granules requires membrane fusion between lytic granules and the PM. An increasing number of studies suggest that small GTPases, including Rab–GTPases, known to regulate membrane trafficking and fusion are important regulators of lytic granule exocytosis in NK cells (191, 192). Following GDP to GTP exchange, these small GTPases are able to interact with their effector proteins involved in protein sorting, motor activity, and tethering, thereby impacting membrane trafficking. Therefore, they are suggested to play important roles, at least in determining specificity of the initial tethering step (Figure 2) (193, 194). In fact, mutation in Rab27a is known to be responsible for a PID known as Griscelli syndrome type 2 (GS2), which will be discussed in detail below (195, 196).

In addition to Rab proteins, the soluble *N*-ethylmaleimide-sensitive factor attachment protein receptor (SNARE) proteins, which are known to mediate most membrane fusion events in eukaryotic cells, also participate in exocytosis (197–199). There are approximately 40 SNARE family proteins in humans with different combinations of SNARE proteins expressed in different cell types. In addition, each SNARE protein exhibits a distinct localization pattern suggesting that selective pairings of SNARE members in specific organelles (like lytic granules) constrain their target binding (197–199). All SNARE proteins contain a coiled-coil SNARE motif at the center, and are structurally categorized as either an R-SNARE or Q-SNARE (further subclassified as Qa-, Qb-, Qc-, or Qb,c-SNARE). Membrane fusion is induced when an R-SNARE protein, such as those found on the lytic granule membrane binds to its cognate Q-SNARE proteins expressed on the PM (Figure 2). Bundling of four SNARE motifs (one from the R-SNARE, and three motifs from two or three Q-SNAREs) makes a stable SNARE complex, and the exerted mechanical force pulls the two membranes closer to promote membrane fusion. Interestingly, familial hemophagocytic lymphohistiocytosis type 4 (FHL4) is the result of a mutation in the gene encoding a Qa-SNARE protein called syntaxin-11 (STX11) (200, 201). In addition to the selectivity of SNARE proteins, accessory proteins including SM (Sec1/Munc18-like), Munc13-like, and synaptotagmin associate with SNARE proteins to regulate membrane fusion both temporally and spatially (197–199, 202). Mutations in Munc13-4 and Munc18-2/syntaxin-binding protein 2 (STXBP2) are responsible for familial hemophagocytic lymphohistiocytosis type 3 (FHL3) and familial hemophagocytic lymphohistiocytosis type 5 (FHL5), respectively (Figure 2) (203–205). Lastly, the entry of extracellular Ca^2+^ is essential for lytic granule exocytosis (206). Although PIDs regarding this category are not discussed in this review (please refer to reviews (21, 207) for more details), NK cells from patients deficient in either the ER calcium sensor STIM1 or the PM calcium channel ORAI1 showed defective cytotoxicity due to impaired exocytosis of lytic granules, although polarization of lytic granules toward the CS was normal (206). Significantly, Munc13 proteins and synaptotagmin proteins contain two calcium-dependent phospholipid-binding C2 domains and are known to regulate activity of the SNARE complex in a Ca^2+^-regulated manner (197, 199, 202). Likewise, members of the synaptotagmin-like protein (Slp) family also share C-terminal tandem C2 domains. Slps were also shown to interact with activated Rab27 (Rab27–GTP) via the Slp homology domain (SHD) and participate in docking granules to the membrane (208). Unfortunately, we still do not have a clear picture as to which of these proteins are regulating the membrane fusion steps in NK cell-mediated cytotoxicity. However, as discussed below, clues to understanding this seemingly complicated and tightly regulated mechanism of lytic granule docking and fusion have been uncovered by functionally antagonizing these molecular pathways and examining NK cells from PID patients deficient in these key molecules.

### Griscelli syndrome type 2

Griscelli syndrome type 2 is an autosomal recessive human disease caused by mutations in the *RAB27A* gene that encodes the small GTPase Rab27a (195, 196). GS2 is clinically characterized by severe immunodeficiency, an accelerated phase of HLH, partial albinism, and distinctive silvery-grayish hair (209, 210). GS2 is distinguished from other subtypes of GS (GS1: mutation in *MYO5A* encoding myosin Va; GS3: mutation in *MLPH* encoding melanophilin) from the fact that only GS2 is associated with the development of hemophagocytosis (211). Since all three responsible proteins for GS are required for distribution of melanins in melanocytes (212), hypopigmentation due to defective melanosome function is a common feature of all subtypes of GS. On the other hand, the fact that cytotoxic lymphocytes only express Rab27a among the three proteins explains why abnormal immune function is only observed in GS2 (213). NK cells from GS2 patients showed defective cytotoxicity which could be at least partially restored by treatment of IL-2 (192, 214–217). In CTLs of GS2 patients as well as Rab27a mutant *Ashen* mice, docking of lytic granules at the PM and the subsequent degranulation process were impaired (195, 204, 218, 219). Supporting the role of Rab27a observed in CTLs, degranulation of activated NK cells from GS2 patients was defective (192), and depletion of Rab27a in the human NK cell-line, NKL, showed a decreased number of lytic granules at the PM (Figure 2) (191). As discussed above, synaptotagmin-like proteins 1–3 (Slp1–3), which are effectors of Rab27a, are expressed in CTLs and involved in lytic granule exocytosis (177, 220, 221). Interestingly, it was recently found that Slp3 interacts with the kinesin-1 motor protein. This is significant, as the motor activity of kinesin-1 was suggested to deliver the slp3/Rab27a complex toward the CS of CTLs (177). Lastly, while Rab27a is in a distinct compartment from lytic granules in both resting NK cells and CTLs (192, 222), when they are activated, Rab27a is recruited to lytic granules. Whether Rab27a binding to Slp3 or some other effector molecule mediates this recruitment remains to be determined.

### Familial hemophagocytic lymphohistiocytosis type 3

Familial hemophagocytic lymphohistiocytosis type 3 is caused by mutation in *UNC13D*, which encodes Munc13-4 (204). Like other types of FHL, FHL3 patients present with hyperactive CTLs and macrophages in the peripheral blood. Patients usually have fever, hepatosplenomegaly, defective coagulation, and features of hemophagocytosis (204, 223, 224). Initial studies of Munc13-4 function reported that Munc13-4 is highly expressed in hematopoietic cells and involved in cytotoxicity of CTLs by mediating the exocytosis of lytic granules (204). In line with this finding in CTLs, cytotoxic activities (both natural cytotoxicity and ADCC) were defective in NK cells from FHL3 patients, whereas levels of cytokines produced by activated NK cells were normal (48, 53, 225). Consistent with the defect being at the level of lytic granule fusion, the polarization of lytic granules toward the CS was normal, but the patient NK cells failed to degranulate (Figure 2) (53, 54). As indicated above, Munc13-4 contains one diacylglycerol (DAG) binding C1 domain and two Ca^2+^-binding C2 domains (204), suggesting it regulates SNARE conformation in Ca^2+^-dependent manner like other Munc13 members in a process called “priming,” which guides incomplete SNARE components for full assembly. However, it is still unclear which target SNARE proteins are regulated by Munc13-4 and how it acts mechanically during NK cell degranulation. However, one of the interesting findings to note is that Munc13-4 was found to be an effector of activated Rab27a (Rab27a–GTP) (226, 227). When NK cells become activated, both proteins were found to associate with lytic granules, and the recruitment of each protein was dependent on the other (192). It is also very intriguing to note that the recruitment of these proteins to lytic granules also requires myosin IIA, since inhibition of myosin activity blocked their recruitment (192). Clearly much more work is needed to delineate the mechanisms by which Rab27a and Munc13-4 regulate lytic granule docking and fusion.

### Familial hemophagocytic lymphohistiocytosis type 4

Familial hemophagocytic lymphohistiocytosis type 4 is caused by mutations in the gene that encodes the Qa-SNARE protein STX11 (200, 201). STX11 is considered an atypical member of the syntaxin family, since it lacks the hydrophobic transmembrane domain found in other syntaxin family members that is involved in membrane binding (228). Instead, STX11 is able to associate with membranes via a cysteine-rich region located at its C-terminal end (229). NK cells from FHL4 patients or human NK cells depleted of STX11 showed defects in both natural cytotoxicity and specific activating receptor-mediated cytotoxicity (54, 230). Polarization of lytic granules toward the CS was normal in these NK cells, but they failed to degranulate suggesting the roles of STX11 in the late step of lytic granule release (Figure 2). Recently established STX11-deficient mice also support findings from the patients (231, 232). Interestingly, the NK cell defects from FHL4 patients were partly rescued with IL-2 treatment (54). It is of interest that STX11 localizes to the late endosome and the TGN (233, 234). Over-expressed STX11 also showed a distinct localization pattern that was different from both Rab27a and perforin in resting NKLs, but upon activation, they colocalized with each other at the CS. It is unclear why these proteins (Rab27a, Munc13-4, and STX11) exist in distinct subcellular compartments prior to activation, but one possible explanation would be the separation of exocytic machineries and lytic granules in the resting state to prevent premature granule exocytosis. Another important issue remaining to be resolved is the identification of the other SNARE complex members that contribute to membrane fusion. Two R-SNARE proteins, VAMP4 and VAMP7, were recently shown to colocalize with perforin in activated NK cells, and mediate lytic granule exocytosis (235, 236). The Qb,c-SNARE protein, SNAP23, is known to interact with STX11 in B cells; therefore, it is intriguing to hypothesize that it might also mediate degranulation of lytic granules in NK cells in cooperation with STX11. STX11 was also identified to interact with Munc18-2/STXBP2, which might play important roles in priming the SNARE complex containing STX11 (203, 205).

### Familial hemophagocytic lymphohistiocytosis type 5

Familial hemophagocytic lymphohistiocytosis type 5 is a recently identified type of FHL caused by mutation in *STXBP2*, which encodes Munc18-2 (also called STXBP2) (203, 205). FHL5 patients have either a very low level of Munc18-2 or no expression due to homozygous or compound heterozygous mutations in the *STXBP2* gene. In addition to common clinical features of FHL, some FHL5 patients additionally presented with colitis, bleeding disorders, and hypogammaglobulinemia (210, 224). Munc18-2 is a member of the SM (Sec1/Munc18-like) protein family, and likely guides appropriate SNAREs for productive complex formation (197–199, 202). Interestingly, Munc18-2 was found to colocalize and interact with STX11 and the protein expression level of STX11 seems to correlate with that of Munc18-2. However, the absence of STX11 did not affect Munc18-2 protein levels suggesting that Munc18-2 might be the main SM protein regulating the STX11 SNARE complex (203). Importantly, NK cells from these patients presented with impaired cytotoxic activity due to defective degranulation (Figure 2) (203, 205, 224, 237). Interestingly, similar to what has been observed in NK cells from FHL4 patients, IL-2 stimulation of NK cells from FHL5 patients partially rescued the cytotoxicity defects, suggesting that the IL-2 pathway is able to bypass the defective Munc18-2/STX11 pathway in both FHL4 and FHL5.

## Conclusion

Advances in clinical diagnostics has substantially increased the identification of patients with PIDs that affect NK cell numbers or effector functions. In addition to furthering our understanding of NK cells in the immune system (CKND and FKND), this group of diseases has substantiated the important roles that NK cells play in immune surveillance. Furthermore, the quantitative and/or qualitative defects in NK cells derived from these patients have highlighted essential molecular machineries shared among immune cells and their importance in NK cell-mediated cytotoxicity. In fact, these loss-of-function mutations have provided invaluable insight into the molecular processes regulating the development of cell-mediated killing by NK cells. However, there are many issues that still remain unanswered. First, how can IL-2-mediated signaling pathways restore NK cell functions in multiple PIDs? Secondly, what are the NK cell-specific mechanisms that differentiate the cytotoxic processes engaged in NK cells from those of CTLs (from biogenesis to exocytosis of lytic granules)? Lastly, how do the signaling molecules that regulate the detailed secretory pathway of lytic granules in NK cells cooperate with the multiple tethering and fusion molecules to promote lytic granule exocytosis? It is clear that PIDs have substantially impacted our understanding of NK cell biology and the cellular mechanisms that control the tightly regulated process of lytic granule release. Further mechanistic insight into the process of NK cell-mediated killing will hopefully reveal novel therapeutic approaches to treat PID patients.

## Conflict of Interest Statement

The authors declare that the research was conducted without any commercial or financial relationships that could be construed as a potential conflict of interest.
